# Segmental paleotetraploidy revealed in sterlet (*Acipenser ruthenus*) genome by chromosome painting

**DOI:** 10.1186/s13039-015-0194-8

**Published:** 2015-11-19

**Authors:** Svetlana A. Romanenko, Larisa S. Biltueva, Natalya A. Serdyukova, Anastasia I. Kulemzina, Violetta R. Beklemisheva, Olga L. Gladkikh, Natalia A. Lemskaya, Elena A. Interesova, Marina A. Korentovich, Nadezhda V. Vorobieva, Alexander S. Graphodatsky, Vladimir A. Trifonov

**Affiliations:** Institute of Molecular and Cellular Biology SB RAS, Novosibirsk, Russia; Novosibirsk State University, Novosibirsk, Russia; Novosibirsk Branch of the Federal State Budgetary Scientific Institution “State Scientific-and-Production Centre for Fisheries (Gosrybcenter)”, Novosibirsk, Russia; Tomsk State University, Tomsk, Russia; Federal State Budgetary Scientific Institution “State Scientific-and-Production Centre for Fisheries (Gosrybcenter)”, Tyumen, Russia

**Keywords:** Acipenseriformes, Fish cell line, Banding, Satellite DNA, Telomeric repeat, rRNA, FISH, Microdissection

## Abstract

**Background:**

Acipenseriformes take a basal position among Actinopteri and demonstrate a striking ploidy variation among species. The sterlet (*Acipenser ruthenus*, Linnaeus, 1758; ARUT) is a diploid 120-chromosomal sturgeon distributed in Eurasian rivers from Danube to Enisey. Despite a high commercial value and a rapid population decline in the wild, many genomic characteristics of sterlet (as well as many other sturgeon species) have not been studied.

**Results:**

Cell lines from different tissues of 12 sterlet specimens from Siberian populations were established following an optimized protocol. Conventional cytogenetic studies supplemented with molecular cytogenetic investigations on obtained fibroblast cell lines allowed a detailed description of sterlet karyotype and a precise localization of 18S/28S and 5S ribosomal clusters. Localization of sturgeon specific HindIII repetitive elements revealed an increased concentration in the pericentromeric region of the acrocentric ARUT14, while the total sterlet repetitive DNA fraction (C_0_t30) produced bright signals on subtelomeric segments of small chromosomal elements. Chromosome and region specific probes ARUT1p, 5, 6, 7, 8 as well as 14 anonymous small sized chromosomes (probes A-N) generated by microdissection were applied in chromosome painting experiments. According to hybridization patterns all painting probes were classified into two major groups: the first group (ARUT5, 6, 8 as well as microchromosome specific probes C, E, F, G, H, and I) painted only a single region each on sterlet metaphases, while probes of the second group (ARUT1p, 7 as well as microchromosome derived probes A, B, D, J, K, M, and N) marked two genomic segments each on different chromosomes. Similar results were obtained on male and female metaphases.

**Conclusions:**

The sterlet genome represents a complex mosaic structure and consists of diploid and tetraploid chromosome segments. This may be regarded as a transition stage from paleotetraploid (functional diploid) to diploid genome condition. Molecular cytogenetic and genomic studies of other 120- and 240-chromosomal sturgeons are needed to reconstruct genome evolution of this vertebrate group.

## Background

A great interest in the study of the sturgeon genomes (Acipenseridae, Acipenseriformes) is primarily connected with a high commercial value of the representatives of the family and a necessity in conservation measures due to a rapid population decline in the wild. At present, most of sturgeons became commercially valuable and popular objects of industrial farming. A detailed investigation of sturgeon biology including molecular characterization of chromosomal complement and understanding of genetic mechanisms of sex determination are essential for improvement of aquaculture and development of a viable conservation strategy. The group of Aciperseriformes also draws attention due to a basal position within Actinopteri on the evolutionary tree of ray-finned fishes. Deep investigation of sturgeon’s genomes is critical for eliciting information about genetic composition through comparative approach.

Despite a high interest in sturgeon biology, the phylogenetic relationships between species, the number of chromosomes and other important biological characteristics remained controversial for a long time. Recent work on sturgeon phylogeny finally resolved many questions [1–3]. However, the cytogenetic investigation of sturgeons was particularly complicated because of the high number of chromosomes in acipenserid karyotypes (a minimal diploid number is about 120). The average diploid number chromosomes in Acipenseriformes considerably exceeds that in other vertebrate groups due to presumed ancient polyploidization event with no diploid ancestral forms survived [4]. Sturgeons’ karyotypes were investigated only by conventional cytogenetics and no molecular chromosome probes were developed for in-depth study of sturgeons’ chromosome structure. The same reasons resulted in the lack of accurate knowledge about the system of sex determination of all members of Acipenseriformes.

However, through pioneering cytogenetic studies of sturgeons karyotypes some essential information about composition and molecular structure of sturgeon chromosomal complements is available [5]. A considerable amount of work on conventional cytogenetics was carried out on other acipenserid species, as well as the study of distribution of telomeric sequences, 5S, 18S, and 28S ribosomal RNA genes, different satellite DNA sequences by fluorescent in situ hybridization (FISH) [6–8]. Up to now the description of some chromosome rearrangements was obtained for only one sturgeon species – *Acipenser gueldenstaedtii* [7].

The sterlet (*Acipenser ruthenus*) is one of the well-known representatives of Acipenseridae family with a relatively wide distribution (from Danube to Enisey) and small body size (in comparison to other sturgeons). The species is considered as vulnerable by the IUCN but it was successfully bred in captivity and sterlet fishing is currently allowed in some Russian regions. The mechanism of sex determination is not established in acipenseriformes, while some existing data suggest genetic sex determination with females being heterogametic in certain species [9–11]. Cell cultures were obtained for sterlet previously which advanced the species cytogenetics [12]. The data on sterlet karyotype description obtained up to 1999 are summarized in [5]. The most recent data show that even the question about precise diploid chromosome number remains open; with 2n reported to vary between 118 ± 2 and 118 ± 4 (see [13]). It was proposed that the sterlet genome, along with other acipenserid genomes with 2n ≈ 120, was formed by duplication of the ancestral 60-chromosomal genome [13]. Other cytogenetic data for *A. ruthenus* include information about С-banding [14], NORs visualization by Ag-staining [4, 15], localization of telomeric repeats [7], detection and mapping of 18S/28S and 5S rRNA [8, 16], and distribution of HindIII satellite [17]. GTG (G-banding by trypsin using Giemsa) differential staining as well as comparison of different markers localization between males and females has not been reported. The comparative information about distinguishing features of male and female karyotypes is also missing. Most cytogenetic works have been accomplished on captive individuals or involved European sterlet populations, no karyotypes of wild sterlet from Siberian rivers were reported so far.

Although chromosome painting using chromosome specific probes was found to be a method of choice for contemporary cytogenetic studies of mammals [18], birds [19], reptiles [20] and even some teleosts [21], no such studies have been performed so far within the group of sturgeons.

Generation of detailed cytogenetic maps saturated with molecular and cytogenetic markers is a prerequisite for a profound study of any genome. However quality metaphases and high-resolution chromosomes are required for reliable localization of molecular probes and for distinguishing of individual chromosome pairs. Here we established an array of sterlet primary cell lines and present a molecular cytogenetic study of sterlet karyotype from Siberian populations using C- and G-banding, localization of variety of repetitive sequences (telomeric repeats, 18S/28S and 5S rDNAs, repetitive DNA fraction (C_0_t 30), and HindIII satellite). Besides, through microdissection we created molecular markers for some of the sterlet chromosomes and applied chromosome painting to male and female metaphases to estimate to copy numbers of homologous regions. We explore and discuss ploidy phenomenon in the sterlet.

## Results

### Optimization of cell culture conditions for primary cell lines of sterlet

To optimize conditions for sterlet cell lines establishment, fin tissues from 5 specimens from the wild population of Ob river (Middle Ob, Tomsk region) (ARUT”1-5”) (Table 1) were used. Cell proliferation was observed in all culturing conditions but growth rates varied. We compared cell growth from explants that undergone collagenase/hyaluronidase proteolytic treatment and those simply plated onto culturing surface. New cell growth was observed after one to three days following seeding of tissue explants regardless of whether proteolytic treatment of explants was performed or not. The cells demonstrated rapid growth and formed a monolayer after seven-ten days of culturing. In all cases fin-derived cells appeared to look better and grew faster if the cultures were established without tissue treatment with proteolytic enzymes. We also compared an array of media: αMEM, DMEM, RPMI, L-15, and 199. The worst results of growing were shown with L-15 medium, the best results was achieved using 199 medium or αMEM supplemented with 15 % FBS. This optimal media combination was validated on fin tissues from ARUT”6-9” individuals and was applied in all subsequent experiments. Moreover, we revealed that sterlet cells are sensitive to standard trypsin/EDTA treatment, therefore we used scrapers to dissociate cells. Post-recovery survival of cells frozen in plain FBS with 10 % DMSO was much higher than for cells frozen in medium with 40 % FBS + 10 % DMSO. In primary sterlet cultures we observed a high viscosity of the post-culture media that decreased with subsequent passaging. This phenomenon is worth additional investigation and could possibly be caused by changes in hyaluronic pathway in sterlet cells similar to that described for the naked mole rat cells [22].

**Table 1:** List of *Acipenser ruthenus* specimens

Abbreviation	Sex	Age	Origin
ARUT”1f”	♀	3–4 years	Shegarsky district, Ob river, N 56°34’45”, E 84 °10’46”, Tomsk oblast, Russia
ARUT”2m”	♂	3–4 years	Shegarsky district, Ob river, N 56°34′45″, E 84 °10′46″, Tomsk oblast, Russia
ARUT”3m”	♂	3–4 years	Shegarsky district, Ob river, N 56°34′45″, E 84 °10′46″, Tomsk oblast, Russia
ARUT”4f”	♀	3–4 years	Shegarsky district, Ob river, N 56°34′45″, E 84 °10′46″, Tomsk oblast, Russia
ARUT”5f”	♀	3–4 years	Shegarsky district, Ob river, N 56°34′45″, E 84 °10′46″, Tomsk oblast, Russia
ARUT”6f”	♀	4 years	Kostylevo, Sturgeon Hatchery Farm of State Scientific-and-Production Centre for Fisheries, Tyumen, Russia
ARUT”7m”	♂	4 years	Kostylevo, Sturgeon Hatchery Farm of State Scientific-and-Production Centre for Fisheries, Tyumen, Russia
ARUT”8f”	♀	4 years	Kostylevo, Sturgeon Hatchery Farm of State Scientific-and-Production Centre for Fisheries, Tyumen, Russia
ARUT”9m”	♂	4 years	Kostylevo, Sturgeon Hatchery Farm of State Scientific-and-Production Centre for Fisheries, Tyumen, Russia
ARUT”10m”	♂	unknown	Fish Farm, Seversk, Tomsk oblast, Russia
ARUT”11f”	♀	unknown	Fish Farm, Seversk, Tomsk oblast, Russia
ARUT”12f”	♀	unknown	Fish Farm, Seversk, Tomsk oblast, Russia

**Fig. 1:**
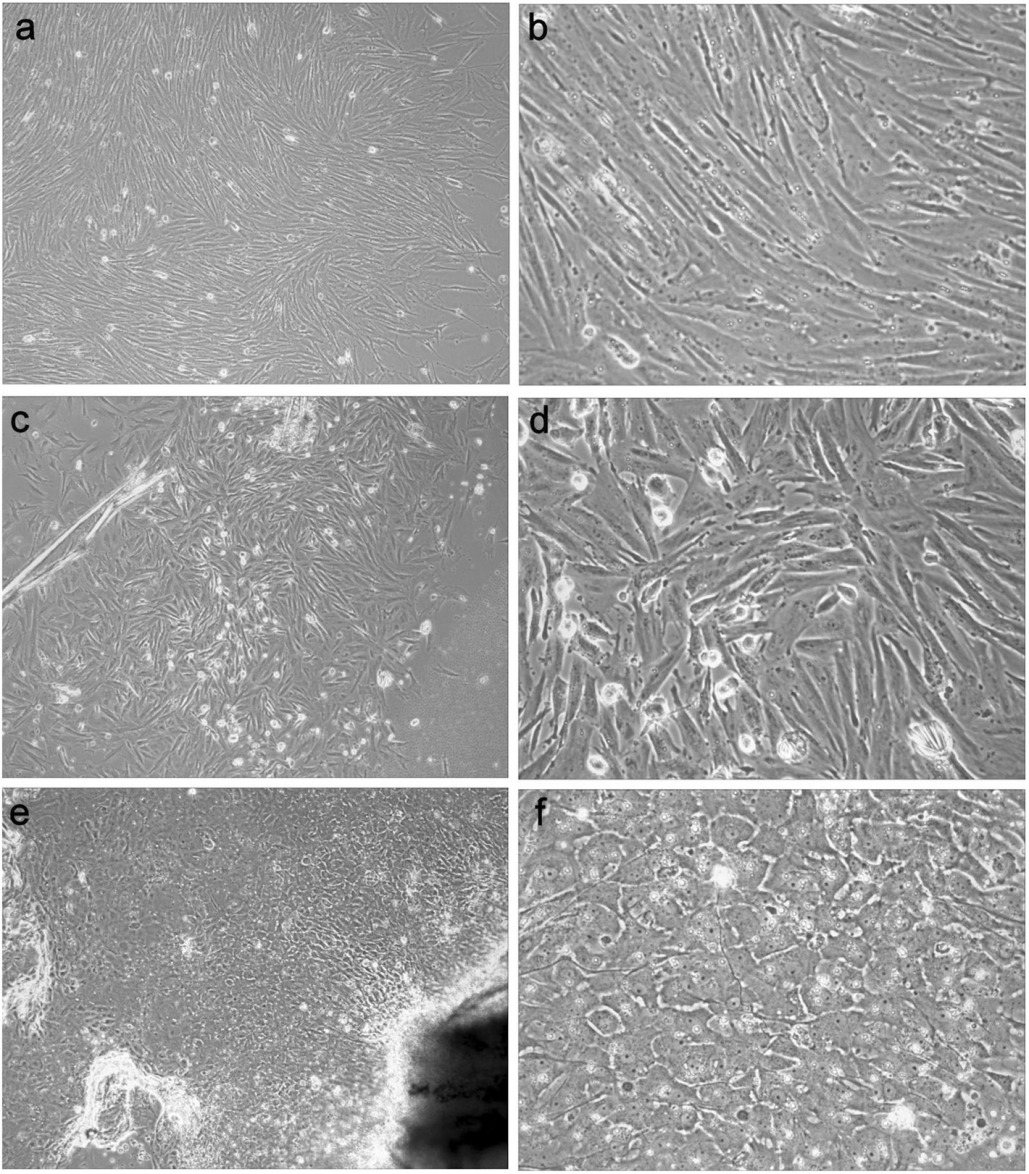
The variety of cell types in primary cultures of sterlet. Left column – 100-fold magnification, right column – the same area at 400-fold magnification. **a**, **b** – сell cultures established from notochord of the male sterlet (ARUT”10m”); **c**, **d** – сell cultures established from swim bladder of the female sterlet (ARUT”11f”); **e**, **f** – сell cultures established from fin of the female sterlet (ARUT”12f”)

In another experiment besides fins we took three different kinds of tissues from ARUT”10-12”: notochord, swim bladder, and barbels and observed similar pattern of growth despite variation in cell morphology (Table 2, Fig. 1). While the cells originated from swim bladder and notochord showed typical fibroblast-like morphology, other cell lines were heterogeneous. We postulate that seeding without enzyme treatment can be more efficient in the case of fin tissues, while notochord tissues demonstrate better growth after preliminary treatment with collagenase and hyaluronidase in comparison to seeding without any treatment. Both methods of swim bladder seeding gave similar results. The establishment of sterlet cell cultures from barbel tissues looked unpromising because of the high risk of contamination and a poor survival of cells after passaging.

**Table 2:** Types of seeding and culture media used for ARUT”10-12” cultivation

Tissues	Without enzymes	With collagenase and hyaluronidase
Barbels	αMEM	-
	199	-
Notochord	αMEM	αMEM
	199	199
Swim bladder	αMEM	αMEM
	199	199
Fin	αMEM	-
	199	-

The optimal condition for each sample is underlined

### Conventional cytogenetics

**Fig. 2:**
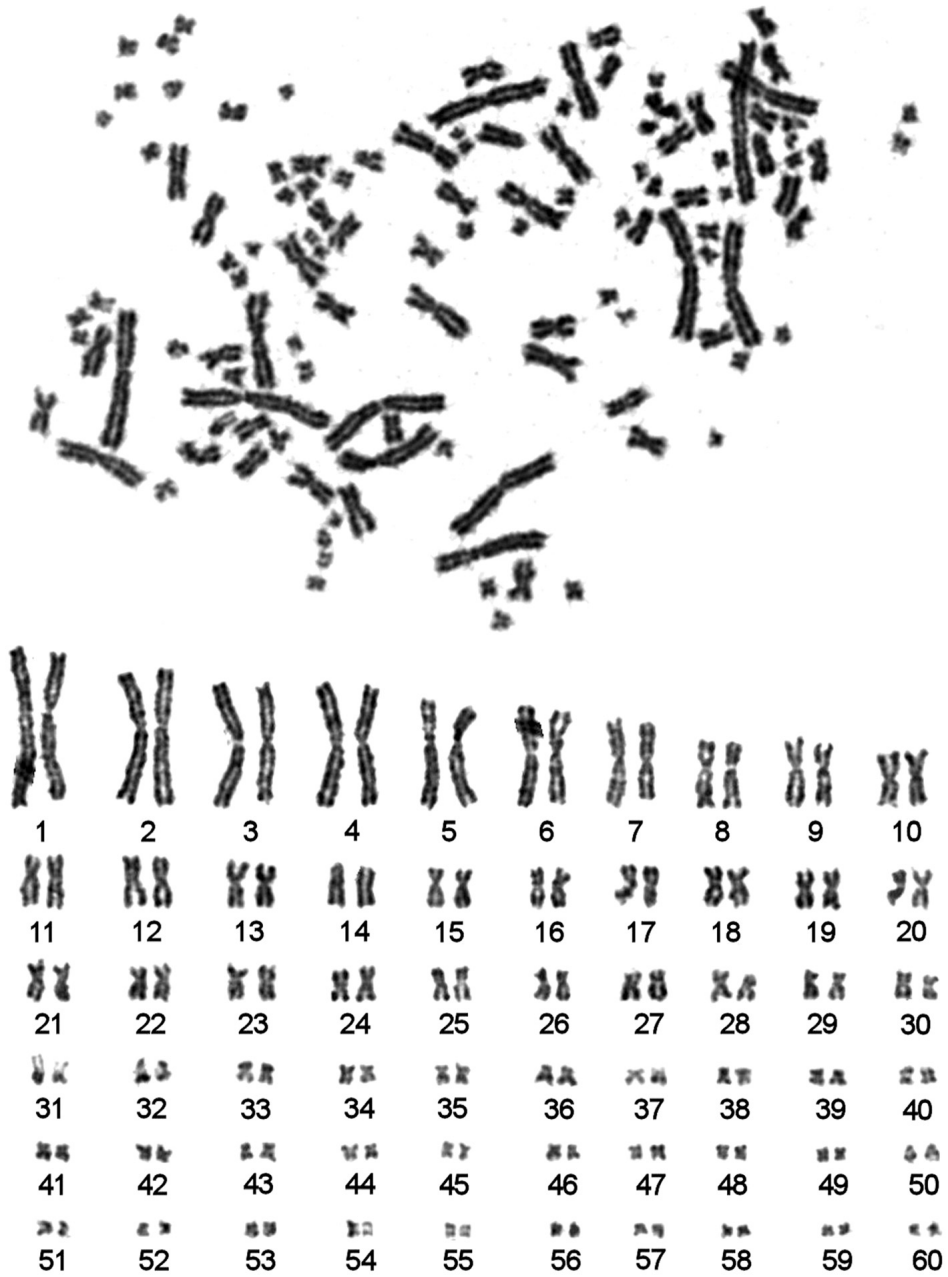
A metaphase plate and karyotype of the male sterlet (ARUT”2m”, 2n = 120) after routine Giemsa staining

**Fig. 3:**
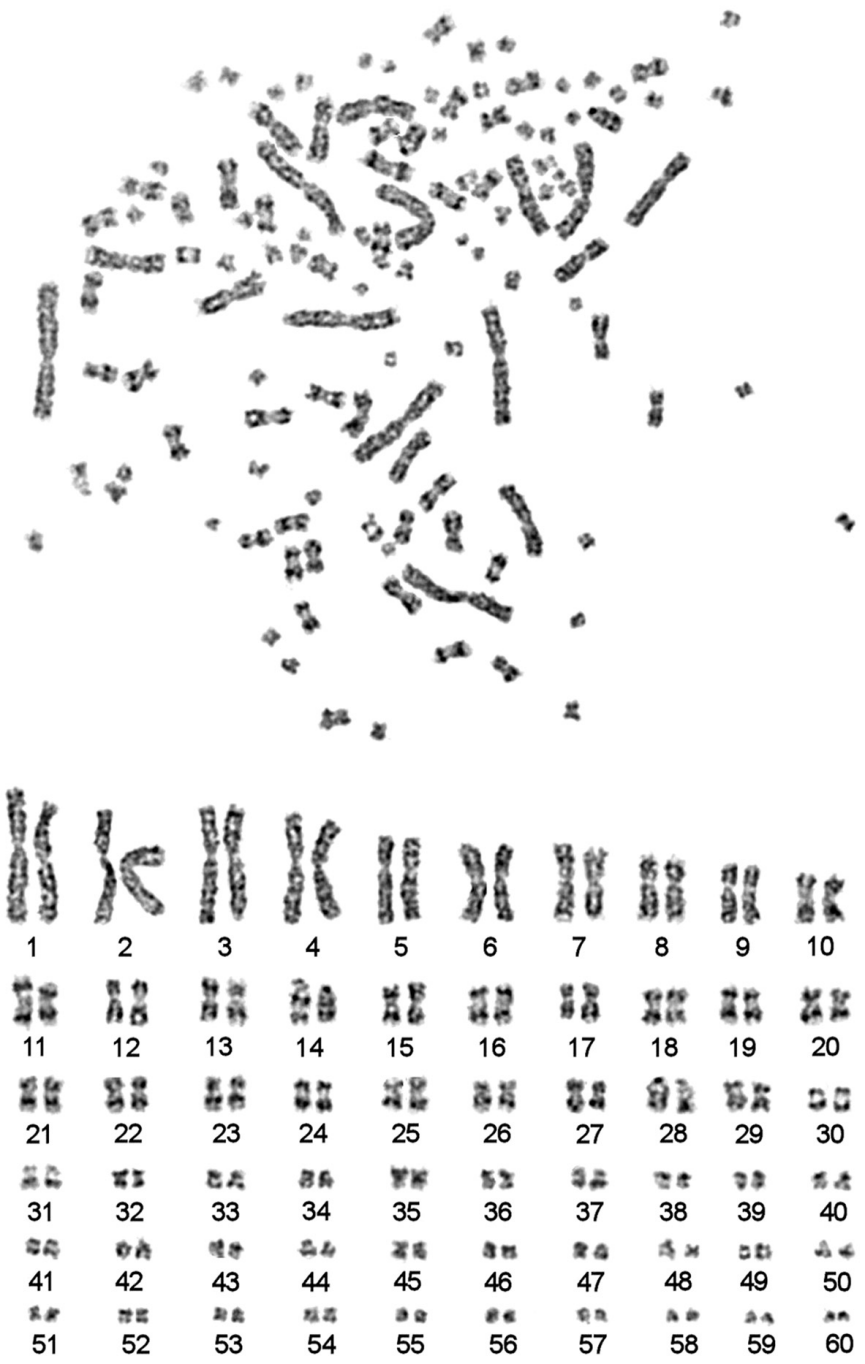
GTG-banded chromosomes of the male sterlet (ARUT”2m”): metaphase plate and karyotype

**Fig. 4:**
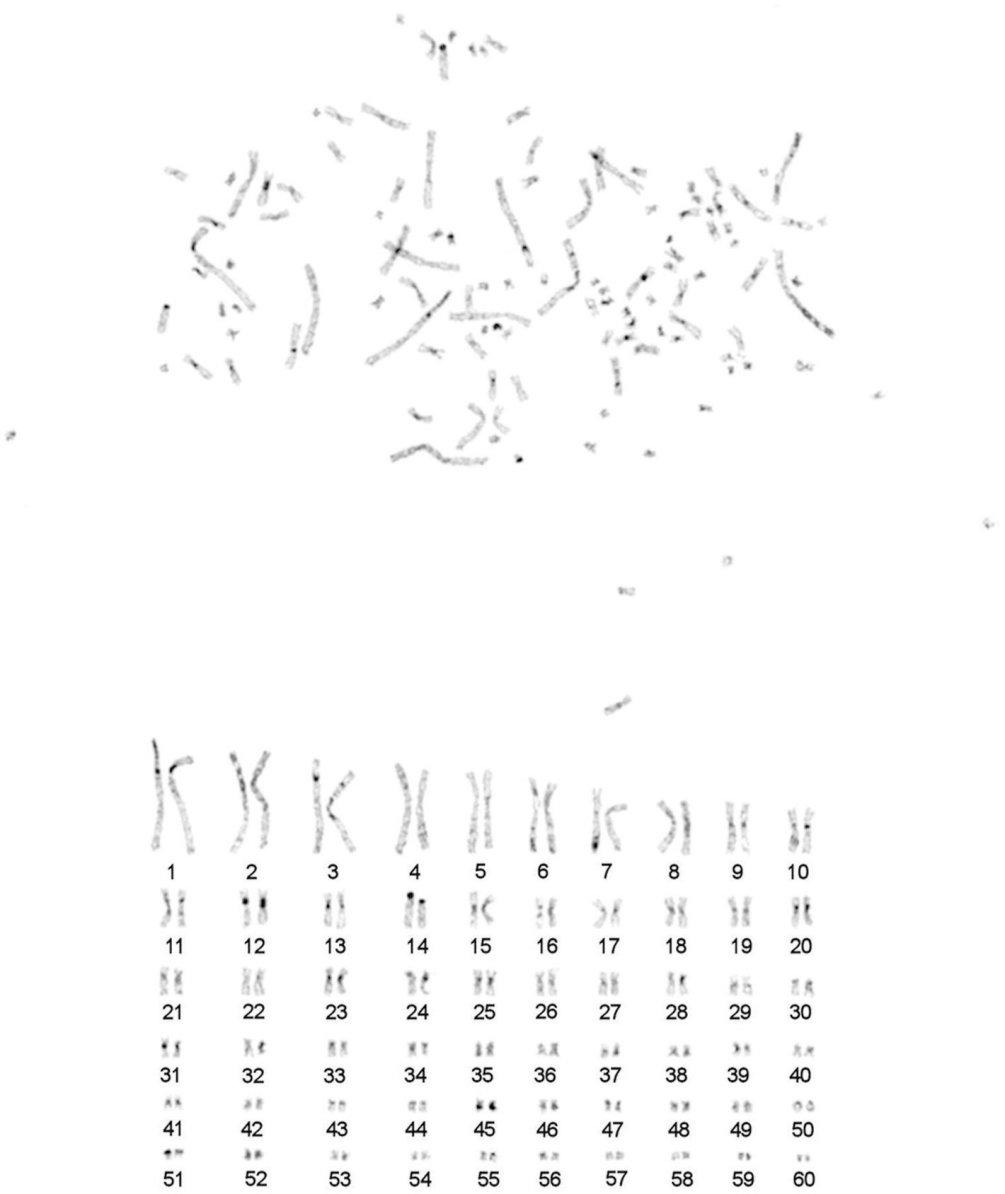
C-banding of sterlet female (ARUT”6f”) chromosomes

Routine Giemsa staining was used to count chromosomes. It appears that 2n in sterlet karyotype is seemingly 120 (Fig. 2). GTG-banding allowed us to further rank chromosome pairs. All pairs of autosomes were placed in order of decreasing size. No distinct G-blocks were identified on large chromosomes (Fig. 3). Heterochromatic blocks were identified in the pericentromeric regions of some sterlet chromosomes (Fig. 4). The largest eight pairs of chromosomes exhibit only interstitial heterochromatin blocks with almost no detectable C-blocks in the centromeric regions of chromosomes.

### Distribution pattern of telomeric repeats and ribosomal DNA

**Fig. 5:**
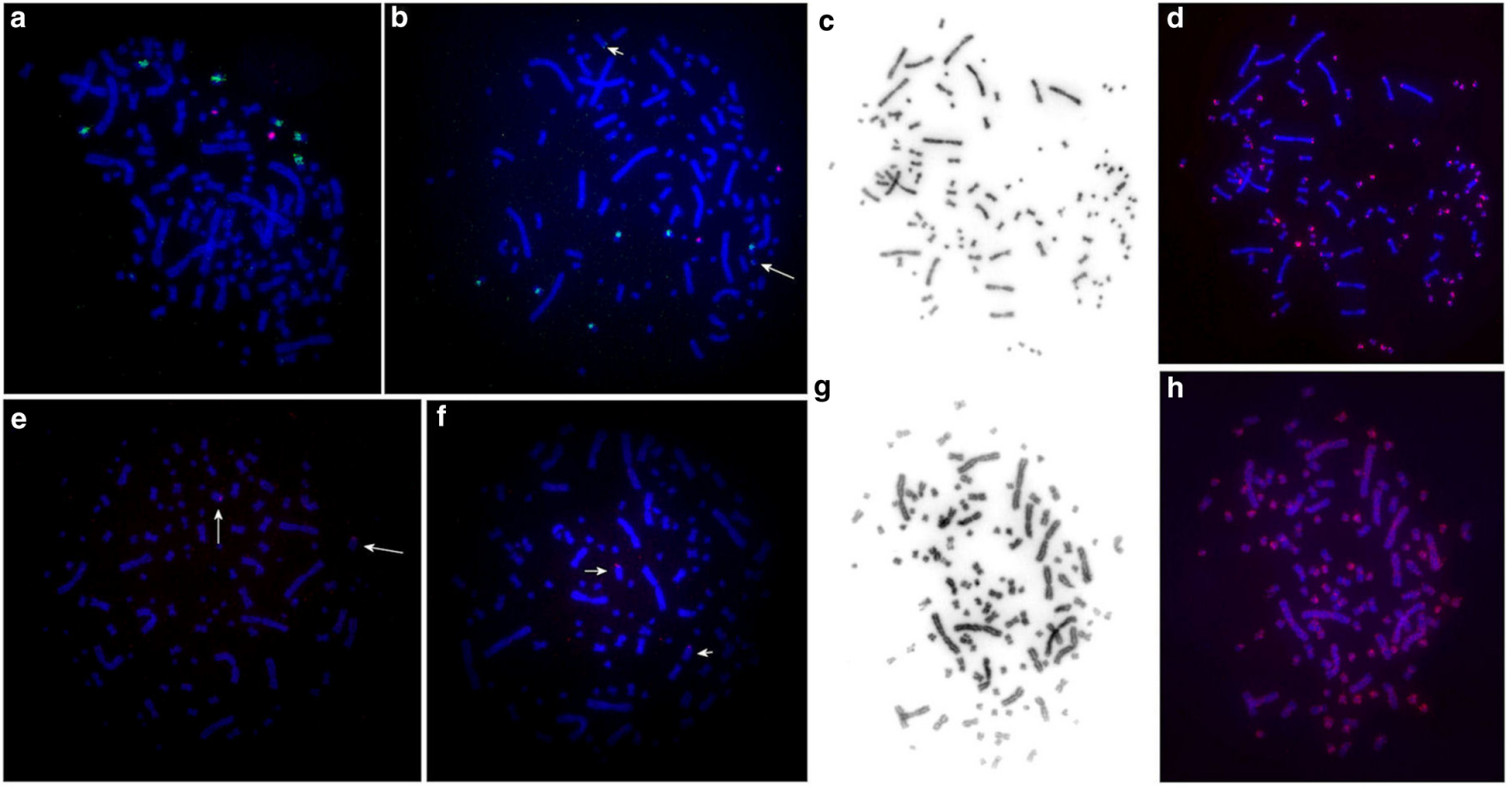
Fluorescent in situ hybridization (FISH) of repetitive DNA probes: **a**, **b** – dual-color FISH with 18S/28S-rDNA probe (*green*) and 5S-rDNA probe (*red*) onto sterlet male and female, respectively; arrows mark some weak additional signals on female chromosomes; **c** – inverted-DAPI image of a male chromosome metaphase spread; **d** – localization of telomeric DNA probe (*red*) onto the same metaphase; **e**, **f** – HindIII satellite onto male and female, respectively; **g** – inverted-DAPI image of a female chromosome metaphase spread, **h** – localization of labeled C_o_t30 DNA (*red*) onto the same metaphase

We localized the 18S/28S-rDNA probe in dual-color FISH with 5S-rDNA probe both on sterlet male and female (Fig. 5a, b). The 5S-rDNA probe marked a pericentromeric region of one of the small pairs of chromosomes in both sexes (ARUT41-50). The pair was DAPI-positive. The 18S/28S-rDNA probe gave 3 pairs of signals on male karyotype: in the p-arms of one pair of chromosomes (ARUT21-30), on the pericentromeric region of a small pair (ARUT31-40) and on the long arm of a small pair of chromosomes (ARUT31-40) (Fig. 5a). Moreover in the female karyotype we detected some additional weak signals produced by 18S/28S-rDNA probe (Fig. 5b). On average we identified from two to four additional signals on different homologs (usually only on a single homolog from the pair). Telomeric repeats were localized in the terminal regions of all chromosomes. Although no interstitial blocks of telomeric repeats were visualized, some small chromosome had increased subtelomeric signals (Fig. 5e, f).

### Distribution pattern of C_o_t30 DNA and HindIII repeat

In all metaphases of both male and female of *A. ruthenus* the hybridization signals with the HindIII satellite DNA probe were weak but clearly visible (Fig. 5c, d). In both sexes the satellite DNA was localized in the pericentromeric region of the large acrocentric pair (ARUT14). No clear signals were detected on other chromosomes.

C_o_t30 DNA probe has highlighted pericentromeric regions of all chromosomes as well as some interstitial regions and p- and q-arms of most small metacentrics (Fig. 5g, h). Signal intensity was higher on small chromosomes suggesting uneven distribution of repetitive DNAs across genome.

### Chromosome painting of microdissection-derived painting probes

**Fig. 6:**
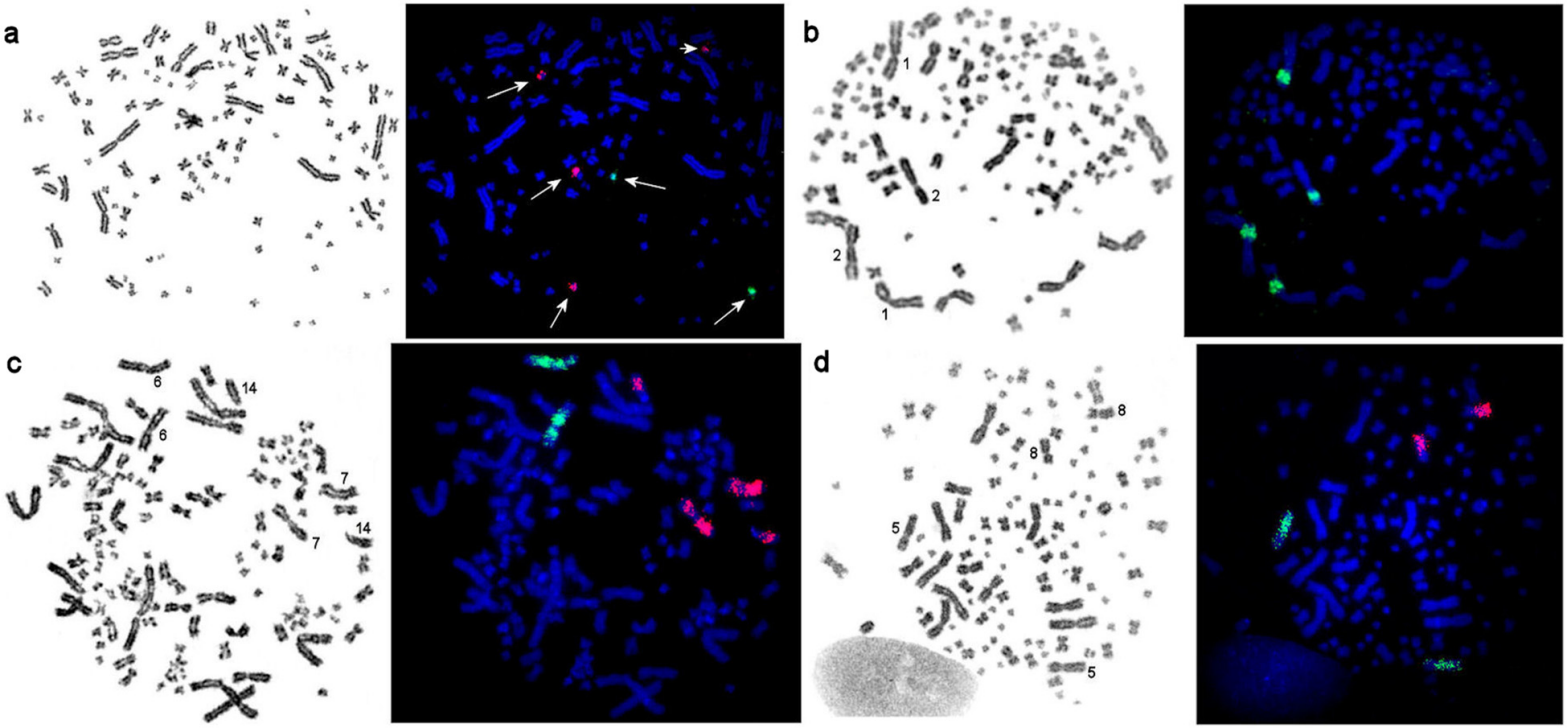
FISH of microdissection-derived painting probes: **a** – painting probes ARUT”A” (*red*) and ARUT”G” (*green*) mark 4 and 2 chromosomes, respectively, on metaphase plate of sterlet female; **b** – painting ARUT1p marks p-arms of chromosomes ARUT1 and ARUT2 in sterlet female; **c** – painting probes ARUT6 (*green*) and ARUT7 (*red*) mark 2 and 4 homologous regions, respectively, in sterlet female; **d** – painting probes ARUT5q (*green*) and ARUT8q (*red*) paint a single chromosome pair each in sterlet male

We obtained painting probes from single chromosomes (regions) ARUT1p, 5, 6, 7, 8 as well as for 14 small sized chromosomes (probes A-N: we used letters to designate the probes of microchromosomes as no precise chromosome assignment had been accomplished yet). All probes obtained can be classified into two major groups: the first group (ARUT5, 6, 8 as well as microchromosome specific probes C, E, F, G, H, and I) painted only a single region each in sterlet genome (Fig. 6a (green signals), c (green signals), d), while probes of the second group (ARUT1p, 7 as well as microchromosome derived probes A, B, D, J, K, M, and N) marked two genomic segments each on different chromosomes (Fig. 6a (red signals), b, c (red signals)). Similar results were obtained on male and female metaphases, revealing no sex specific localization pattern.

It is interesting to note that the most tetraploid segments were localized on chromosomes of similar size (i.e., ARUT1p and ARUT2p), but the probe ARUT7 additionally painted much smaller and different in morphology chromosome ARUT14. We assume that some material of ARUT7 is present in diploid and some in tetraploid state.

## Discussion

### Features of sterlet cell culture

Cell cultures of different sturgeon species have been established since 1985 [23]. Previously published data showed that fish cell cultures can be grown using variety of culture media [24–27]. Fin tissues are the most commonly used material for establishing primary cell line in fishes. However, we show that primary cell line could be established successfully from variety of sterlet tissues types (notochord, bladder). In present work we used the established growth temperature and FBS concentration shown to be optimal for other sturgeon species [26, 27]. We varied several parameters of culturing and show that proteolytic treatment is very efficient for establishment of primary cell lines from notochord, but not useful for fins [28, 29]. We demonstrated that 199 and alphaMEM media are suitable for prolific cell growth in sterlet cell lines, while L-15 media is not (Table 2). Sterlet cells from all tissues are sensitive to trypsin and freezing, so extra measures should be taken to not damage the cells by harsh handling. Scrapers and mild composition of cryopreservation media (90 % FBS + 10%DMSO) should be used to keep cells alive through standard cell line procedures. Successful application of this protocol for another sturgeon species (*A. baerii*) (unpublished data) indicates that this method can be used for tissue culture establishment from variety of sturgeon species.

### Karyotype of the sterlet

Cytogenetic description of the sterlet karyotype have been published previously (e.g., [5, 14, 30]).

The karyotype of *A. ruthenus* is very similar to karyotypes of other 120-chromosomal acipenserids. Most of the chromosomes are bi-armed, whereas two pairs (ARUT14 and ARUT50) are acrocentric. Previously, the presence of at least two pairs of acrocentric chromosomes was described by Rab [30]. At the same time, on the basis of routine staining authors could not establish unambiguously morphology of small pairs of chromosomes (ARUT32-60). Establishment of cell cultures and optimization of harvesting protocol allowed us to obtain metaphase chromosomes with a high resolution and to characterize the morphology of small chromosome pairs as bi-armed.

As it was previously shown the largest eight pairs of chromosomes contain only interstitial heterochromatin blocks with almost no visible C-blocks in the pericentromeric regions (Fig. 4, [14]). Precise pair-by-pair comparison of data obtained here with published previously is complicated by the lack of standard nomenclature. Many microchromosomes of *A. ruthenus* were previously described as almost or totally heterochromatic [14, 31], while here we show that most of these chromosomes contain euchromatic regions. Based on present data, clearly visible C-bands were detected at pericentromeric regions of chromosomes ARUT9-10, 12–14, 20, 23, 25, 31 and 51. Only two microchromosomes ARUT45 and 59 contained large stretches of heterochromatin and no visible euchromatic components.

It is noteworthy that no distinct and reproducible G-block pattern was detected in the sterlet karyotype, which is usually observed in the karyotypes of warm-blooded vertebrates and some fishes [32]. Previously a parallelism between chromosome banding and compositional compartmentalization of fishes genome was supported [32]. We suggest that the absence of reproducible G-banding pattern in sterlet might also be result of a compositional homogeneity of its genome.

### Distribution pattern of telomeric repeats and ribosomal RNA genes

The telomeric repeated sequence (TTAGGG)_n_ is highly conserved in structure and function among eukaryotes [33]. At the moment the sequence has been localized in over 100 vertebrate species, including fishes. In *A. ruthenus* karyotype the telomere signals were detected by FISH as definite spots at both ends of each chromosome, although the signal intensity varied between chromosomes [7]. Rather large blocks of repeats have been found on some small metacentrics. We could not find any variation in telomeric repeats distribution between male and female specimens. In general telomeric blocks distribution in studied here sterlet individuals is similar to previously published for wild and captive populations [7].

Different cytogenetic approaches reveal specific features of nucleolar organizing regions (NORs). Interesting that sturgeon NORs were not stained by GC specific fluorochromes as in other groups fishes studied [31]. While conventional Ag-staining reveals only active NORs, FISH with rDNA probes detects all clusters of rDNA, regardless of their activity. Using Ag-staining NORs were detected at the terminal ends of two chromosome pairs in sterlet [4, 15]. Localization of rRNA genes in *A. ruthenus* by FISH with the 28S and 5S probes yielded signals at 3 and 1 pairs of chromosomes, respectively [16]. Later studies revealed from 6 to 8 chromosomes (3–4 pairs) as NOR-bearing using FISH with the 18S/28S probe [8]. Moreover, the authors mentioned that all 5S rDNA signals overlapped with some of the 18S/28S rDNA signals [8]. In the present study we revealed unusual features in 18S/28S-rDNA probe distribution in two studied individuals (ARUT”9m” (male) and ARUT”12f” (female)) (Fig. 5a, b). In the female specimen three pairs of intense signal were detected (common with male), but additionally, we observed some weak signals on different chromosomes (Fig. 5b). The number of the additional signals varied from 2 to 4. Moreover in some cases we could clearly identify only one of the homologs bearing NOR. As for now only one individual of each sex was investigated and most likely that such pattern of 18S/28S-rDNA probe localization points out at individual variation, although it also could result from heteromorphism of some *A. ruthenus* chromosomes. Additional investigation of NOR localization in more male and female individuals is needed. The amount of signals revealed using 5S-rDNA probe in the male and female was the same as published previously [16]. Dual-color FISH did not show any overlapping between 5S and 18S/28S-rDNA probes localizations (Fig. 5a). The discrepancy between the results obtained here and previously published could be attributed to variation between sterlet populations.

### Distribution patterns of C_o_t30 DNA and HindIII repeat

Satellite DNA is an important component of eukaryotic genome, mostly composed of tandemly repeated nucleotide sequences. The satellite DNA does not encode proteins and is localized in the regions of constitutive heterochromatin, preferentially in pericentromeric and subtelomeric areas of chromosomes [34]. The pattern of distribution of different kinds of satellite DNA sequences is one of the distinguishing features of species karyotype. Previous studies of satellite DNA sequence distribution in sturgeons included description of HindIII and PstI enriched heterochromatic blocks in some acipenserid species [17, 35].

In earlier studies the HindIII satellite DNA probe revealed minimum 8 signals on chromosomes of sterlet [17]. In our samples HindIII repetitive DNA was localized in the pericentromeric region of only one chromosome pair (the large acrocentric ARUT14) in both sexes and we did not detect any clear signals on other chromosomes (Fig. 5c, d). Such variation in the amount of signals obtained here and in the previous work could point out at a variation of HindIII satellite DNA content and chromosomal distribution between populations. The amount and the size of HindIII specific blocks indicate that it is not the major component of sterlet heterochromatin.

C_o_t DNA fraction is rich in numerous types of repetitive sequences and isolation of the repetitive DNAs was proved to be useful for genome characterization in many animal and plant species [36–39]. Depending on the fraction C_o_t DNA contains various amounts of satellite DNAs, DNA transposons, and retrotransposons. In some species localization of C_o_t DNA onto metaphase chromosomes could produce a banding pattern, useful for chromosome identification [38]. We isolated C_o_t30 fraction of DNA that includes wide range of repetitive elements. Physical mapping of the C_o_t30 probe in *A. ruthenus* karyotypes revealed repeat-rich blocks on both arms of all small chromosomes except for their pericentromeric regions (Fig. 5g, h). On the contrary, a higher intensity of signals was detected in pericentromeric regions of large chromosomes, but signals were diffused. Generally, the variation in the pattern of C_o_t30 DNA distribution between small and large chromosomes revealed here could indicate the repeat homogeneity of chromosomes inside these two groups and will help in future development of chromosome specific markers.

### Partial tetraploidization of sterlet genome

In his classical work S. Onho has proposed that genome evolution might have been accompanied by polyploidization events [40]. Modern genomic studies largely confirm this hypothesis and provide evidence that whole genome duplication events were quite common in the past and are characteristic for different eukaryotic taxa [41]. Interestingly some animal groups (such as mammals and birds) seem to be highly intolerant to genome duplications (or even to partial chromosome segment duplication). Although it was proposed that the 102-chromosomal genome of the South American red vizcacha rat (*Tympanoctomys barrerae*) resulted from tetraploidization [42], subsequent chromosome painting data clearly demonstrated that all chromosomal segments are present in diploid state [43]. On the other hand, chromosome painting turned out to be very useful in confirmation of triploidy in some lizards [44]. As most fish genomes have not been involved yet in chromosome painting experiments, future molecular cytogenetic works may shed light onto the level of ploidy in their genomes.

It was proposed that all modern 120-chromosomal acipenserids represent functional diploids, originated over 200 million years ago by a whole genome duplication of a 60-chromosomal ancestor [13, 45, 46]. The transition between 120-chromosomal tetraploid to modern functional diploid might have been accompanied by a functional reduction [2, 7, 15, 47]. Here using chromosome painting we present a direct evidence of partial genome tetraploidy combined with partial diploidy in the same species genome for the first time. It is noteworthy that most chromosomes and chromosomal regions were found to be in either diploid or in tetraploid state. However it is notable that chromosome 7 seems to consist of two blocks – tetrapoid (which also paints chromosome 14) and diploid (paints only a part of chromosome 7) (Fig. 6c). Of course we cannot exclude that the sterlet genome may contain additional highly divergent copies of regions revealed as diploid in FISH experiments. It is interesting, that tetraploidy of 120-chromosomal paddlefish was first proposed by Dingerkus and Howell on the basis on karyotype analysis [48], but later the accumulation of data on molecular markers [15] provided evidence for a diploid state of 120-chromosomal sturgeons and paddlefish. Here we demonstrate that both these hypotheses are partly correct and the genomes of sturgeons might be more complex than it was proposed earlier. Whole genome sequencing is urgently needed to resolve multiple questions regarding the structure and origin of sterlet genome.

## Conclusion

Genome evolution of Acipenseriformes is characterized by many independent polyploidization events on the one hand and by relatively low rates of molecular evolution the other hand [49]. Still very little is known about fundamental issues of sturgeon biology related to genetic mechanism of sex determination, predisposition to polyploidization and interspecific hybridization, genome composition and evolution. The establishment of sterlet cell cultures allowed us to obtain high quality chromosome preparations for molecular cytogenetic experiments including FISH, chromosome microdissection and chromosome specific painting probe generation. Chromosome painting revealed a complex structure of sturgeon genome comprising regions with different ploidy levels and indicated that further work is necessary to estimate precisely the ratio between diploid and tetraploid genomic components. Besides, we did not find any sex specific hybridization patterns among probes obtained here assuming that the search for sex chromosomes should be continued by means of the construction of more chromosome specific markers and comparative genome sequencing.

## Methods

### Ethics statement

The protocol was approved by the Committee on the Ethics of Animal Experiments of the Institute of Molecular and Cellular Biology SB RAS. Sterlet individuals were incubated in the water with 10^−4^ (v/v) Eugenol for about 5 min for euthanasia. All efforts were made to minimize suffering.

### Samples origin

In total 12 sterlet specimens (6 males and 6 females) originating from Ob (ARUT”1-5”), Irtysh (ARUT”6-9”) and Enisey (ARUT”10-12”) rivers were provided for study by State Science-and-Production Centre for Fisheries (Table 1).

### Optimization of sterlet cell culture conditions

To find optimal conditions for sterlet cell lines establishment, tissues from 5 specimens from the wild population of Ob river (Middle Ob, Tomsk region) (ARUT”1-5”) were used.

Before dissecting fish was patted with dry paper towel to remove mucus. Abdominal and pectoral fins were additionally wiped with 70 % ethanol. Fins and surrounding tissue were cut and incubated in 70 % ethanol for 3 min. Subsequent manipulations were made in a sterile environment. Fins were rinsed out twice in 199 medium with penicillin (5*10^5^ U/L), streptomycin (500 mg/L) and amphotericin B (12.5 mg/L) and incubated overnight in a fresh portion of the same medium. All samples were cultivated in CO_2_ controlled incubator (5 % CO_2_) at 25 °C, in each case growth medium contained 15 % of FBS, penicillin (1*10^5^ U/L), streptomycin (100 mg/L) and amphotericin B (2.5 mg/L).

We used two protocols for cell culture establishment. Some samples were digested by proteolytic enzymes (collagenase and hyaluronidase) to dissociate tissue and release individual cells, other samples were attached to flask surface without preliminary treatments. The modified protocol of tissue culture establishment without enzymes was described earlier [50]. In both variants all five types of culture media (αMEM, DMEM, RPMI, L-15 and 199) were used for cells cultivation.

### Establishment of cell cultures using collagenase/hyaluronidase treatment of tissues

We used the protocol suggested by Stanyon and Galleny [28] for mammalian tissues with some modification. The tissue pieces were finely minced and placed in a tube with 1–2 ml collagenase/hyaluronidase mixture: 1 mg/ml collagenase, 1 mg/ml hyaluronidase, 15 % fetal bovine serum in the growth medium. Dispersed tissues were incubated in CO_2_ controlled incubator for 24 h at 25 °C. After that the pelleted cells were placed in culture flask with the growth medium.

### Cell line passaging

For sequential passages the cells were dissociated with 0.25 % tripsin, 0.2 % EDTA or taken off by scrapers.

### Application of the optimal conditions for establishment of cell cultures from notochord, swim bladder and barbels

The optimized conditions were applied for establishment of additional cell lines from 7 specimens (ARUT”6-12”) from fishery farms of Tyumen Oblast and Tomsk Oblast. We took some other tissues (notochord, swim bladder and barbels) for cultivation (Table 2). Notochord and swim bladder tissue were asceptically removed for culturing. Barbles were cleaned and immersed as fins in 70 % ethanol for 3 min.

### Cryopreservation and thawing of cells

We applied two different protocols for freezing cells. In the first protocol we used 199 medium with 40 % FBS and 10 % DMSO, in the second we used plain FBS with 10 % DMSO for freezing. The cryovials were placed in CoolCell (BioCision) freezing container and stored at −72 °C overnight. Cryovials were then transferred into cryotank with liquid nitrogen (−196 °C) for a prolonged storage.

For recovery, vials were thawed in the water at 30 °C, cells were then resuspended in 5X volume of 199 medium with 15 % FBS and centrifuged at 0,6× g for 5 min. After removing the supernatant, the cells were resuspended in the 199 medium with 15 % FBS, then counted in Goryaev’s chamber using trypan blue stain (most cells appeared alive upon staining) and seeded into cell culture flasks to estimate the number of survived cells.

### Chromosome preparation

Cells were split at a ratio 1:2 in a medium with 5-10 % of AmnioMax (Gibco). After two days of culturing colcemid (KaryoMAX, Gibco) was added to a final concentration of 0.1 μg/ml for overnight incubation. Three hours before cell harvesting ethidium bromide was added to a final concentration of 1.5 μg/ml. Cells were dissociated mechanically by scraping and centrifuged for 5 min at 0.6× g. Cell pellet was gently resuspended in hypotonic solution (33.5 mМ KCl, 7.75 mM sodium citrate) and incubated for 2 h at 25 °C. For prefixation treatment 1/20 volume of fresh ice-cold fixative (methanol: acetic acid - 3:1) was added, mixed carefully and incubated for 12 min at 4 °C. Then cells were centrifuged for 5 min at 0.6× g and supernatant was discarded. For cell fixation, the pellet was covered by ice-cold fixative (−20 °C) and kept for 30 min at −20 °C without mixing. Cells were then centrifuged for 5 min at 0.6× g and resuspended in ice-cold fixative. The chromosome suspensions were stored long-term at −20 °C.

### Chromosome staining

Routine Giemsa staining, C- and G-banding were performed as described previously [51].

### Telomeric and ribosomal DNA probes

The telomeric DNA probe was generated by PCR using the oligonucleotides (TTAGGG)_5_ and (CCCTAA)_5_ [52]. Clones of human ribosomal DNA containing the complete 18S-rRNA and 28S-rRNA genes were obtained as described [53] and labeled by nick translation following the manufacturer’s protocol (Nick Translation System, Life Technologies). 5S-rDNA probe was amplified by PCR using following primers: 5′-TACAGCACTTGATATTCCCA-3′ and 5′-GTCATGAAAGCAGAAATGCA-3′. 5S-rDNA PCR amplification was performed in a 100 μl reaction mixture, containing 65 mМ Tris–HCl (pH 8.9), 16 mМ (NH_4_)_2_SO_4_, 2.5 mM MgCl_2_, 0.05 % tween-20, 0.25 mМ 3dNTP, 0.1 mМ TTP, 0.1 mМ dig-dUTP, 400 ng of sterlet genomic DNA, 2 U of Taq DNA-polymerase, 1 μМ of each primer. The PCR protocol included denaturing at 94 °C for 2 min, 30 cycles of denaturing at 94 °C for 30 s, annealing at 58 °C for 30 s and extension at 72 °C for 1 min 20 s. Agarose gel electrophoresis was performed to estimate the size of the PCR product (~100 bp).

### HindIII satellite probe

The probe of HindIII satellite previously described by De la Herrán et al. [49] was obtained by PCR using primers: 5′-TTGATCTTCAGAACTACCAA-3′ and 5′-GGAACGAACCTGTAAGCTT-3′. PCR amplification was performed in 100 μl reaction mixture, containing 65 mМ Tris–HCl (pH 8.9), 16 mМ (NH_4_)_2_SO_4_, 2.5 mM MgCl_2_, 0.05 % tween-20, 0.25 mМ 4dNTP, 0.08 mМ dig-dUTP, 400 ng of sterlet genomic DNA, 2 U Taq DNA-polymerase, 1 μМ of each primer. PCR protocol included denaturation at 94 °C for 2 min, 35 cycles of denaturing at 94 °C for 20 s, annealing at 58 °C for 50 s and extension at 72 °C for 1 min 20 s.

### C_o_t fraction of repeated DNA

C_o_t30 DNA was obtained as described previously [54]. Labeling was carried out using Niсk Translation Kit (Sigma). Labeled C_o_t30 DNA was used as a probe for fluorescent in situ hybridization (FISH).

### Painting probe generation by chromosome microdissection

Microdissection was performed as described earlier [55]. DNA from a single copy of each microdissected chromosome was amplified and labelled using WGA kits (Sigma). In total we obtained painting probes from following chromosomes (regions): ARUT1p, 5, 6, 7, 8 as well as for 14 small sized chromosomes.

### Fluorescent in situ hybridization

FISH was performed on freshly made chromosome preparations not subject to any proteinase or RNAse treatment. Hybridization mixture contained 12 μl of 50 % formamide, 2 × SSC, 0.2 % Tween 20, and 0.2 μg of probe. Probes were denatured at 95 °C for 5 min. Slides were incubated in PBS with 0.05М MgCl_2_ for 5 min and then in 2xSSC for 5 min. Chromosome denaturation was done in 70 % formamide with 2xSSC at 67 °C for 30–40 s. FISH protocol was described previously [56]. The slides were analyzed with fluorescence microscopes Olympus BX53 and Axioskop 2 plus (Zeiss) using VideoTesT-Karyo and VideoTesT-FISH (VideoTesT, Saint-Petersburg, Russia) digital imaging systems.
